# The pan-variant potential of light: 425 nm light inactivates SARS-CoV-2 variants of concern and non-cytotoxic doses reduce viral titers in human airway epithelial cells

**DOI:** 10.1128/msphere.00230-25

**Published:** 2025-05-28

**Authors:** Nathan Stasko, Leslee Arwood, Nicole Jandick, Derry Spragion, Rachel C. Roberts, Mónica Setién, Ibrahim Henson, Abigail Annas, M. Leslie Fulcher, Marisa Brotton, Larry Kummer, Frank Szaba, Matt Reagan, Kathleen Lanzer, Tres Cookenham, Sean Casey, Nagarama Kothapalli, Tricia Hart, Shelton S. Bradrick, David Emerson, Adam S. Cockrell, Scott H. Randell, Jacob F. Kocher

**Affiliations:** 1 EmitBio Inc, Morrisville, North Carolina, USA; 2 The Marsico Lung Institute, The University of North Carolina at Chapel Hill, Chapel Hill, North Carolina, USA; 3 Trudeau Institute, Saranac Lake, New York, USA; 4 Department of Cell Biology and Physiology, The University of North Carolina at Chapel Hill, Chapel Hill, North Carolina, USA; Johns Hopkins University Bloomberg School of Public Health, Baltimore, Maryland, USA

**Keywords:** coronavirus, photomedicine, antiviral agents

## Abstract

Severe acute respiratory syndrome coronavirus 2 (SARS-CoV-2) variants of concern (VOCs) prolonged the coronavirus disease 2019 (COVID-19) pandemic. The continued development of novel pan-variant therapeutics to treat currently circulating and future VOCs is critically important. Photomedicine may offer broadly applicable, pan-variant treatments. In this study, we show that visible light centered around 425 nm inactivates each of the five SARS-CoV-2 VOC lineages that have been identified by the World Health Organization (Alpha, Beta, Delta, Gamma, and Omicron) in cell-free suspensions in a dose-dependent manner, including bamlanivimab-resistant variants. Specifically, 60 J/cm^2^ of 425 nm light reduced SARS-CoV-2 titers by >4 log_10_ relative to unilluminated controls. We observed that 425 nm light inactivates SARS-CoV-2 through restricted entry to host cells. In addition, a non-cytotoxic dosing regimen of 32 J/cm^2^ of 425 nm light reduced infectious virus titers in well-differentiated air–liquid interface (ALI) human airway epithelial (HAE) cells infected with the Beta, Delta, and Omicron variants that incorporate mutations associated with immune evasion and/or increased transmissibility. Infectious SARS-CoV-2 titers were reduced when dosing began during the early stages of infection or in more established infections. Finally, we translated these findings to the RD-X19, a novel medical device that emits 425 nm light; our results showed that the RD-X19 restricted spike binding to ACE-2 and reduced SARS-CoV-2 titers in cell-free suspensions (by >2 log_10_) and in the ALI HAE model (by >1 log_10_). These findings indicate that photomedicine utilizing 425 nm visible light may serve as a novel, pan-variant treatment modality for COVID-19.

**IMPORTANCE:**

The continued spread of severe acute respiratory syndrome coronavirus 2 (SARS-CoV-2) has led to the emergence of variants that can evade public health measures, including vaccines and therapeutics. Thus, the continued development of broadly applicable measures to supplement current public health measures and standards of care remains critical. Photomedicine is one such approach. In this study, we show that non-ultraviolet visible light can inactivate each SARS-CoV-2 variant of concern (VOC) by preventing entry to host cells. Furthermore, visible light reduced the amount of virus produced in an infection model of the human airway at multiple stages of infection, demonstrating the antiviral capability of visible light. This study provides preclinical support for the development of visible light to serve as a SARS-CoV-2 countermeasure and warrants further investigation.

## INTRODUCTION

In late 2019, the severe acute respiratory syndrome coronavirus 2 (SARS-CoV-2), the causative agent of coronavirus disease 2019 (COVID-19), was identified in Wuhan, China. The COVID-19 pandemic resulted in over 750 million confirmed COVID-19 cases and over 7 million deaths worldwide as of February 2025 (1). The rampant spread of SARS-CoV-2 led to the emergence of SARS-CoV-2 variants that each initiated waves of disease and economic burden. Specifically, variants of concern (VOCs), which are characterized by (i) more severe disease outcomes, (ii) the need for major public health interventions, and/or (iii) decreased efficacy of available vaccines, had a significant impact in prolonging the COVID-19 pandemic (1–4). To date, the World Health Organization (WHO) classified five VOCs: Alpha, Beta, Delta, Gamma, and Omicron (2). The Omicron VOC has continued to evolve into antigenically distinct sublineages that are independently characterized as variants under monitoring (VUM) or variants of interest (VOI) by the WHO (2). The emergence of VOCs and the continued emergence of Omicron sublineages have resulted in repeat surges of COVID-19 globally (1).

The COVID-19 pandemic spurred the rapid development of countermeasures to combat the novel pathogen, including the development of vaccines, monoclonal antibodies (mAb), antivirals, and diagnostics. Several therapeutics of differing modalities have been approved or were granted Emergency Use Authorization by the US Food and Drug Administration for hospitalized patients and for those at high risk of disease progression to severe COVID-19. Specifically, remdesivir reduced hospitalizations and time to recovery after hospitalization in moderate-to-severe COVID-19; mAb standalone and cocktail therapies demonstrated significant reductions in hospitalization and mortality; the oral antivirals nirmatrelvir/ritonavir and molnupiravir reduced hospitalizations and deaths in mild-moderate COVID-19; and ensitrelvir reduced the time to first negative SARS-CoV-2 test (5–13). However, COVID-19 treatments are limited by a variety of factors, including the requirement for hospitalization or elevated risk of disease progression, the need for intravenous infusion centers, potential mutagenic side effects, complex drug-to-drug interactions, emergence of hypersensitivities and liver toxicities, and susceptibility to evasion by SARS-CoV-2 variants (14–21). As the world turns toward the treatment of COVID-19 as an endemic respiratory infection, the potential for vaccine- or antiviral-resistant variants to emerge increases. To complement existing therapies, the continued development of pan-variant COVID-19 therapeutics remains of paramount importance.

Photomedicine is one potential treatment modality. Multiple studies have demonstrated that visible light is capable of inactivating SARS-CoV-2 in biological matrices and in aerosols (22–25). Furthermore, light reduces SARS-CoV-2 titers in Vero E6 cell infection models (23, 26) and in productive infection models of the human buccal epithelium and large airway epithelium (23, 27). In a randomized, double-blind, sham-controlled early feasibility clinical trial conducted in late 2020, Stasko et al. demonstrated that the RD-X19, a device emitting 425 nm visible light targeted to the oropharynx, reduced the time to sustained symptom resolution (27). Here, we build on these works and report the following: (i) 425 nm light inactivates each of the five SARS-CoV-2 VOCs in a dose-dependent manner; (ii) 425 nm light prevents SARS-CoV-2 entry into host cells; (iii) 425 nm light reduces Beta, Delta, and Omicron titers in well-differentiated models of the human airway epithelium; and (iv) RD-X19 inactivates SARS-CoV-2 and reduces viral titers in productive infection models. This work provides further evidence that photomedicine utilizing 425 nm visible blue light could serve as the foundation for a new pan-variant antiviral against existing and pre-emergent SARS-CoV-2 variants.

## MATERIALS AND METHODS

### Cells and viruses

Vero E6 cells were purchased from the American Type Culture Collection (ATCC; Manassas, VA, USA) and were maintained as previously described (23). Mv 1 Lu (CCL-64), LLC-MK2 (CCL-7.1), and A549 (CCL-185) cells were purchased from the ATCC. Mv 1 Lu and A549 cells were maintained in high-glucose Dulbecco’s modified Eagle medium (DMEM; Sigma-Aldrich #D6429-500mL) supplemented with 10% heat-inactivated FetalCloneII (Cytiva #SH30066.03hi) and 1% antibiotic/antimycotic (Gibco #15240-0620). LLC-MK2 cells were maintained in Medium 199 (Gibco #11-043-023) supplemented with 5% heat-inactivated FetalCloneII, 1% GlutaMAX (Gibco #35-050-061), 1% non-essential amino acids (Gibco #11-140-050), and 1% antibiotic/antimycotic. Vero E6 cells overexpressing human TMPRSS2 were purchased from Sekisui XenoTech (#JCRB1819). Cells were maintained in T2 growth medium (high-glucose DMEM [Sigma-Aldrich #D6429-500mL] supplemented with 10% FetalCloneII [Cytiva #SH30066.03hi], 1% antibiotic/antimycotic [Gibco #15240-0620], and 1 mg/mL G418 [Sigma-Aldrich #G8168-100mL]). All cells were cultured at 37°C and 5% CO_2_.

The human coronavirus OC43 (VR-1558) and human adenovirus type 5 (VR-5) were obtained from the ATCC. OC43 was passaged on Mv 1 Lu cells, and human adenovirus 5 was cultured on A549 cells. Human rhinovirus 1B (HRV-1B) was obtained and cultured as previously described (23). SARS-CoV-2 isolates WA1 (NR-52281, contributed by the Centers for Disease Control and Prevention), Alpha (NR-54000, contributed by Dr. Bassam Hallis), Beta (NR-54009, contributed by Drs. Alex Sigal and Tulio de Oliveira), Gamma (NR-54982, contributed by the National Institute of Infectious Diseases), Delta (NR-55611, contributed by Drs. Richard Webby and Anami Patel), Omicron BA.1 (NR-56461, contributed by Dr. Andrew S. Pekosz), Omicron BA.2 (NR-56520, contributed by the Centers for Disease Control and Prevention), Lambda (NR-55654, contributed by the Centers for Disease Control and Prevention), Omicron XBB.1.5 (NR-59104, contributed by Dr. Andrew S. Pekosz), Omicron EG.5 (NR-59503, contributed by Dr. Andrew S. Pekosz), Omicron JN.1 (NR-59693, contributed by Dr. Viviana Simon), and NY-PV09158/2020 (NR-53516, contributed by Dr. Adolfo Garcia-Sastre) were obtained through BEI Resources, National Institute of Allergy and Infectious Diseases, National Institutes of Health (Bethesda, MD, USA). All viruses were passaged as previously described (23). All work with active, replication-competent SARS-CoV-2 was conducted in a biosafety level-3 (BSL-3) facility, with strict adherence to established safety guidelines at EmitBio or Trudeau Institute.

Well-differentiated air–liquid interface human primary tracheobronchial airway epithelial (ALI HAE) cell cultures were acquired from the Marsico Lung Institute Tissue Procurement and Cell Culture Core facility at the University of North Carolina at Chapel Hill (Donor DD065Q; Chapel Hill, NC, USA). The cells were cultured and differentiated on Millicell CM inserts in UNC air–liquid interface media as previously described (28). Cells were delivered in 12-well plates with agarose embedded in the basal compartment and revived as previously described (23). Cells were allowed to recover for 3–5 days in 2 mL of air–liquid interface medium (28) in 6-well plates prior to experimental initiation. Antiviral assays were conducted as previously described (23).

### Light plaque reduction neutralization test (PRNT)

Virus suspensions were illuminated via the biological light unit (BLU) with the indicated doses of 405, 425, 450, or 500 nm light as previously described (23). Briefly, virus stocks were diluted in minimum essential medium (MEM) (Gibco #11095-080) supplemented with 2% heat-inactivated fetal bovine serum (FBS; Gibco #10082-147) and 1% antibiotic-antimycotic (Gibco #15240-0620). In artificial saliva studies, viral stocks were diluted in artificial saliva BZ108 (BioChemaZone) supplemented with or without riboflavin-5’-phosphate sodium salt (Sigma-Aldrich #R7774) diluted in sterile phosphate-buffered saline (PBS). Diluted virus stock (500 µL) was added to individual wells of a 24-well plate. Virus was illuminated within the BLU at a fixed irradiance (50 mW/cm^2^) over varying exposure times to deliver a total dose (in joules per square centimeter) as above. In the irradiance testing experiments, irradiance and exposure time were modulated to deliver a total dose of 32 J/cm^2^. SARS-CoV-2 and HRV-1B viral titers were enumerated via plaque assay as previously described (23). SARS-CoV-2 WA1 plaque assays were fixed and stained at 4 days post-infection, and SARS-CoV-2 variants were fixed and stained at 5 days post-infection.

Omicron XBB.1.5, Omicron EG.5, and Omicron JN.1 experiments were conducted at Trudeau Institute Contract Research Organization (TICRO; Saranac Lake, NY, USA). Plaque assays were conducted on Vero E6 cells overexpressing ACE-2 and TMPRSS2 with 1.2% microcrystalline cellulose overlay and were developed following 2 days of incubation. OC43 plaque assays were conducted on Mv 1 Lu cells with 1.2% Avicel overlay and were developed following 5 days of incubation. NL63 plaque assays were conducted on LLC-MK2 cells with 1.2% Avicel overlay and were developed following 5 days of incubation. Ad5 plaque assays were conducted on A549 cells with 1.2% Avicel overlay and were developed following 5 days of incubation.

### Monoclonal antibody PRNT

Bamlanivimab was kindly provided by the Vaccine Research Center, National Institutes of Health. Bamlanivimab (mAb LY-CoV555) was diluted to 4 µg/mL and serially diluted 1:2 in SARS-CoV-2 diluent. SARS-CoV-2 stocks were diluted to 250 PFU/50 µL, and 60 µL was added to each antibody dilution (final concentrations of 2 µg/mL to 0.00391 µg/mL). Virus–antibody combinations were incubated for 1 h at 37°C/5% CO_2_ and then titered via plaque assay as above. Plaques were counted for each antibody dilution and for virus input.

### qRT-PCR

Diluted virus stocks (500 µL of 2 × 10^5^ PFU/mL SARS-CoV-2 WA1) were added to individual wells in 24-well plates (total: 1 × 10^5^ PFU/well) and were illuminated in the BLU. Following illumination, viral RNA was extracted from 200 µL of virus suspension using the QIAamp Viral RNA Mini Kit (Qiagen #52904) following the manufacturer’s instructions. Extracted RNA was aliquoted in triplicate and stored at −80°C. Viral RNA copies were quantified using the N1 and N2 primer/probe sets in the 2019-nCoV CDC RUO Kit (Integrated DNA Technologies #10006713) and the TaqMan Fast Virus 1-Step Master Mix (Thermo Fisher #4444432). The 20 µL total reaction volume consisted of 5 µL Master Mix, 1.5 µL N1 or N2 primer/probe, 8.5 µL molecular-grade water, and 5 µL template RNA. Cycling conditions were 50°C for 5 min, 95°C for 20 s, then 40 cycles of 95°C for 3 s and 60°C for 30 s. A standard curve was generated using the synthetic SARS-CoV-2 RNA (ATCC, #VR-3276SD). The standard curve was diluted from 1 × 10^6^ copies/well to 1 × 10^0^ copies/well in molecular-grade water. Reactions were run on the QuantStudio 3 Real-Time PCR System (Applied Biosystems). Data analysis was conducted on the QuantStudio Design and Analysis Software (Applied Biosystems). Two samples in each reaction (N1 and N2) were not detected due to technical error.

### SARS-CoV-2 spike-ACE-2 binding assay

High-binding ELISA 96-well plates were coated with 200 ng of recombinant human ACE-2 protein (Sino Biological #10108-H02H) or human DPP-4 (Sino Biological #10688-H01H) in 100 µL PBS and were incubated at 4°C overnight. Wells were washed 4× with 200 µL PBS with 0.01% Tween 20 (PBST). Plates were blocked with 200 µL blocking buffer (5% FBS in PBST) and incubated at 37°C/5% CO_2_ for 1 h. His-tagged recombinant WA1 S1 (Sino Biological #40591-V08H), Omicron B.1.1.529 S1 (Sino Biological #40591-V08H41), MERS-CoV S1 (Sino Biological #40069-V08H), SARS-CoV-1 S1+S2 (Sino Biological #40634-V08B), NL63 S1+S2 (Sino Biological #40604-V08B), MERS-CoV S1+S2 (Sino Biological #40069-V08B), WA1 spike trimer (Sino Biological #40589-V08H4), Alpha spike trimer (Sino Biological #40589-V08H12), Beta spike trimer (Sino Biological #40589-V08H13), Gamma spike trimer (Sino Biological #40589-V08H23), Delta spike trimer (Sino Biological #40589-V08H10), Lambda spike trimer (Sino Biological #40589-V08H24), Omicron B.1.1.529 spike trimer (Sino Biological #40589-V08H26), Omicron BA.2 spike trimer (Sino Biological #40589-V08H28), Omicron XBB.1.5 spike trimer (40589-V08H45), and Omicron JN.1 spike trimer (Sino Biological #40589-V08H59) were diluted in viral diluent. Diluted spikes (250 µL) were either added to individual wells in a 96-well plate and illuminated with 425 nm light using the BLU or added to the middle four wells of a 48-well plate and illuminated with the RD-X19. Following blocking, the ACE-2-coated plates were washed 4× with PBST as above. Illuminated samples (100 µL) were added in duplicate to ACE-2-coated plates, incubated at 37°C/5% CO_2_ for 90 min, and washed 4× with PBST. Mouse horseradish peroxidase (HRP)-conjugated anti-6× His tag antibody (Thermo Fisher #MA180218) was diluted 1:200 in 5% FBS in PBS; 100 µL of diluted antibody was added to each well. Plates were incubated at 37°C/5% CO_2_ for 60 min and washed 3× with PBST with a final wash with PBS. Room temperature 1-Step Ultra TMB substrate (100 µL, Thermo Fisher #34028) was added to each well. Plates were developed for 15–30 min at room temperature in the dark. The reaction was stopped with 100 µL of 2 M sulfuric acid. Plates were read on a Promega GloMax Discover at 450 and 605 nm. Virus diluent was added to separate ACE-2-coated wells to serve as a negative control. Background readings and negative control readings were removed, and fold change binding was calculated relative to untreated (0 J/cm^2^). Data presented are the result of two independent experiments.

The whole virus-ACE-2 binding assay study was conducted at TICRO. Virus was diluted in viral diluent and illuminated with 425 nm light via the BLU as above. The binding assay was conducted as detailed above.

### Cell entry qPCR

SARS-CoV-2 Beta was diluted to 2 × 10^5^ PFU/mL and illuminated with 425 nm light (0, 15, and 90 J/cm^2^) as above. Vero E6 cells were inoculated with 200 µL of illuminated virus suspension and were incubated for 1 h at 37°C/5% CO_2_. The inoculum was removed, cells were washed 2× with PBS, and 500 µL of culture media (high-glucose DMEM + 5% FBS [Gibco] + 1% antibiotic-antimycotic) was added to each well. Cells were incubated at 37°C/5% CO_2_ and washed 2× with PBS prior to total RNA extraction. Total RNA was extracted at 3 and 24 hours post-infection (hpi) with the RNeasy Mini Total RNA extraction kit (Qiagen). SARS-CoV-2 RNA was detected with the CDC assay kit (IDT) and the TaqMan Fast Virus 1-Step Master Mix (Thermo Fisher) on the QuantStudio3 System (Invitrogen).

### SARS-CoV-2 spike-expressing pseudovirion cellular entry assay

Pseudovirions expressing WA1 (#RSCOV2-SL-2) and Omicron spikes (#RSCOV2-SDB11529L-2) with luciferase reporters were obtained from Cellecta (Mountain View, CA, USA). Vero E6/TMPRSS2 cells were seeded in 96-well plates with T2 growth medium (as above) at 7 × 10^4^ cells/well and were incubated at 37°C/5% CO_2_ overnight. Cell media were added to empty wells to limit the evaporation in the plate. Cells were incubated with MEM (Gibco #11095-080) supplemented with 2% heat-inactivated fetal bovine serum (Gibco #10082-147) and 1% antibiotic-antimycotic (Gibco #15240-0620) containing 0.1% dimethyl sulfoxide (DMSO), E64d (20 µM, Fisher Scientific #501965092), camostat mesylate (100 µM, Fisher Scientific #C2977100MG), or E64D and camostat mesylate for 2 h at 37°C/5% CO_2_. Fifty microliters (>1 × 10^4^ relative light units [RLU]/mL) of WA1 spike-expressing or Omicron spike-expressing pseudovirions was illuminated in clear 96-well plates. After illumination, the media were removed from the Vero E6/TMPRSS22 cells, and 50 µL of illuminated pseudovirions was transferred to each well and incubated at 37°C/5% CO_2_ for 2 h. Following incubation, 150 µL of media containing each inhibitor was added to each well as indicated, and cells were incubated for 72 h at 37°C/5% CO_2_. After 72 h, plates were removed from the incubator to equilibrate to room temperature. The Bright-Glo Luciferase Assay system reagent (Promega #E2610) was prepared following the manufacturer’s instructions. Cell culture media (90 µL) were removed from each well, and 100 µL of Bright-Glo reagent was added to each well of cultured cells. Plates were gently rocked and incubated at room temperature for 2 min. Lysate (100 µL) was transferred from each well to a white 96-well plate. Plates were read on the Promega GloMax Discover using the preprogrammed BrightGlo program. Data are presented as fold change relative to the group that received 0 J/cm^2^ and 0.1% DMSO.

### RNase protection assay

Heat-inactivated SARS-CoV-2 WA1 was acquired from the ATCC (#VR-1986HK). Viral stocks were diluted in MEM (Gibco #11095-080) supplemented with 2% heat-inactivated fetal bovine serum (Gibco #10082-147) and 1% antibiotic-antimycotic (Gibco #15240-0620); 500 µL of the diluted suspension was placed into individual wells of 24-well tissue culture plates and illuminated via the BLU as above. Following illumination, aliquots of each illuminated suspension were treated with (i) diluent only, (ii) 50 U RNase I (Thermo Fisher #AM2294), or (iii) 0.1% Triton X-100 in PBS with 50 U RNase I and incubated at 37°C/5% CO_2_ for 20 min. Viral RNA was extracted via the Qiagen Viral RNA Mini kit (Qiagen #52904) following the manufacturer’s instructions. Viral RNA copies were enumerated via qRT-PCR as above.

### Statistical analysis

Statistical significance was determined using the Mann-Whitney rank-sum test using GraphPad Prism 8. Statistical significance is indicated by * (*P* < 0.05), ** (*P* < 0.01), ***(*P* < 0.001), and **** (*P* < 0.0001).

## RESULTS

### 425 nm light inactivates SARS-CoV-2 variants of concern

**Fig 1:**
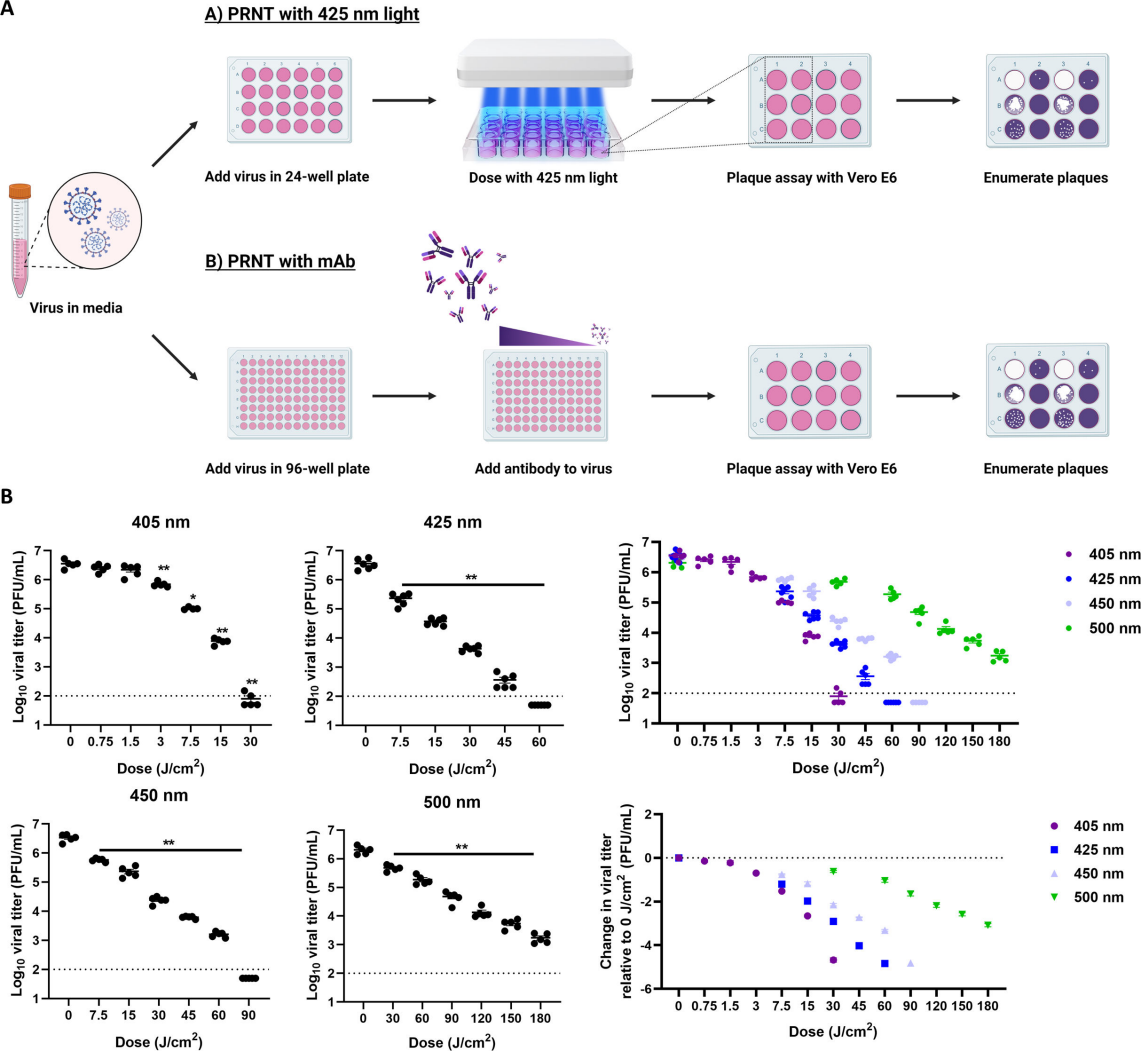
Visible light inactivates SARS-CoV-2 WA1 in a wavelength- and dose-dependent manner. Biological light units were configured to evenly distribute light over the entire surface area of a 24-well plate and used to evaluate various energy densities in the classic PRNT assay method to measure the inactivation of cell-free SARS-CoV-2 (**A**). SARS-CoV-2 WA1 was diluted and illuminated with a fixed dose of visible light per experiment or incubated with serially diluted bamlanivimab prior to infectious virus enumeration with plaque assay. Cell-free suspensions of SARS-CoV-2 WA1 were illuminated with 405, 425, 450, and 500 nm light (**B**). Data presented are mean viral titers (PFU/mL) ± SEM (*n* = 5–6) and viral titer reduction relative to 0 J/cm^2^. Statistical significance was determined compared to the 0 J/cm^2^ group via Mann-Whitney rank-sum test and is indicated by * (*P* ≤ 0.05) and ** (*P* < 0.01). The dotted lines represent the assay limit of detection.

To determine the virucidal activity of different wavelengths of light, we adapted the mAb-based plaque reduction neutralization test and illuminated suspensions of SARS-CoV-2 WA1 with 405, 425, 450, and 500 nm light (Fig. 1A). Visible light significantly inactivated SARS-CoV-2 in a wavelength- and dose-dependent manner (Fig. 1B); however, longer wavelengths of light required higher doses to inactivate SARS-CoV-2. Specifically, 30 J/cm^2^ of 405 nm, 60 J/cm^2^ of 425 nm, and 90 J/cm^2^ of 450 nm light reduced SARS-CoV-2 titer below the assay limit of detection. None of the tested doses of 500 nm light reduced SARS-CoV-2 titer to the assay limit of detection. Based on the similar inactivation kinetics between 405 and 425 nm light and the reduced cytotoxicity of 425 nm light (23), we focused on 425 nm light for further studies.

**Fig 2:**
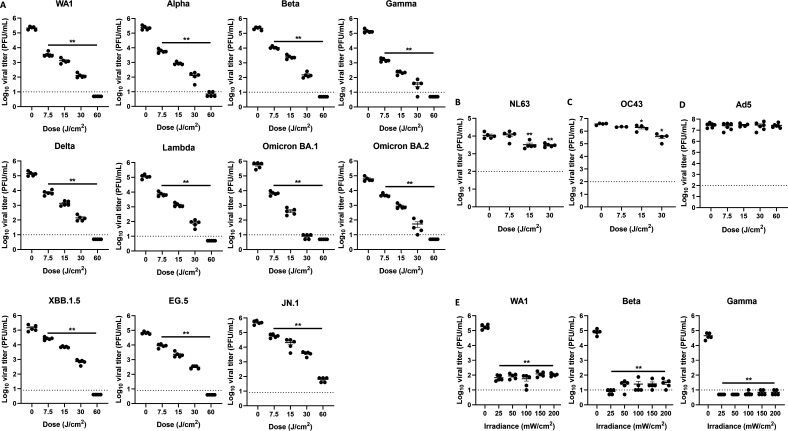
425 nm light inactivates SARS-CoV-2 variants of concern in a dose-dependent manner. The capability of 425 nm light to inactivate SARS-CoV-2 variants of concern was evaluated via the PRNT assay. Cell-free stocks of SARS-CoV-2 variants were diluted and illuminated with a dose range of 425 nm light (**A**). Cell-free stocks of human coronavirus NL63 (**B**), human coronavirus OC43 (**C**), and human adenovirus 5 (**D**) were diluted and illuminated with a dose range of 425 nm light. Cell-free stocks of SARS-CoV-2 variants were diluted and illuminated with a fixed dose of 425 nm light varying by irradiance and administration time (**E**). Viral titers were enumerated via plaque assay. Data presented are mean viral titers (PFU/mL) ± SEM (*n* = 5–6). Statistical significance was determined compared to the 0 J/cm^2^ group via Mann-Whitney rank-sum test and is indicated by * (*P* ≤ 0.05) and ** (*P* < 0.01). The dotted lines represent the assay limit of detection.

To determine whether mutations within SARS-CoV-2 variants impacted inactivation by 425 nm light, we illuminated WA1, four SARS-CoV-2 VOCs (Alpha, Beta, Gamma, and Delta), five Omicron sublineages, and one SARS-CoV-2 variant of interest (Lambda) (Fig. 2) with 425 nm light. In each case, 425 nm light significantly inactivated SARS-CoV-2 variants in a dose-dependent manner (Fig. 2A). For each variant, as little as 7.5 J/cm^2^ of 425 nm light significantly reduced SARS-CoV-2 titer. Except for SARS-CoV-2 Omicron JN.1, 60 J/cm^2^ of 425 nm light reduced the viral titer for each variant to the assay limit of detection. A similar result was observed with an early, non-VOC SARS-CoV-2 variant as well (Fig. S1A). The inactivation kinetics with 425 nm light for WA1, Alpha, Beta, Gamma, Delta, Omicron BA.1, and Omicron BA.2 were comparable (Fig. S1B). Conversely, the mAb bamlanivimab neutralized only WA1 and the Alpha variant; bamlanivimab did not neutralize the Beta or Gamma variants and neutralized only the Delta variant at 2 µg/mL (Fig. S1B and C). The Omicron variants BA.1 and BA.2 also evade neutralization by bamlanivimab (19, 20, 29). These results indicate that 425 nm light has the potential to inactivate SARS-CoV-2 VOCs in cell-free suspensions.

Previous work demonstrated that 425 nm light illumination of suspensions of human rhinovirus 1B (HRV-1B) did not reduce viral titers but did inactivate the highly pathogenic betacoronaviruses Middle East respiratory syndrome coronavirus (MERS-CoV) and SARS-CoV-1 (23). In the present study, we investigated whether 425 nm light inactivated the endemic alphacoronavirus NL63, the betacoronavirus OC43, and the human adenovirus type 5 (hAd5) (Fig. 2B through D). Interestingly, the endemic coronaviruses NL63 and OC43 (Fig. 2B and C) significantly reduced viral titers at 15 and 30 J/cm^2^ but did not demonstrate dose-dependent inactivation as observed with the highly pathogenic coronaviruses. At 30 J/cm^2^, 425 nm light reduced OC43 and NL63 titers by 1 log_10_ and <1 log_10_, respectively, whereas this dose inactivated highly pathogenic coronaviruses by >1 log_10_. Similarly, 425 nm light did not inactivate the non-enveloped endemic virus hAd5 (Fig. 2D). These results illustrate virus-specific illumination by 425 nm light.

Finally, we examined how light irradiance and dosing time impacted 425 nm light inactivation of SARS-CoV-2. To do this, we illuminated SARS-CoV-2 suspensions with 425 nm light at different irradiances and lengths of time to deliver a total dose of 32 J/cm^2^. The irradiances and times tested ranged from 25 mW/cm^2^ for 1,280 s to 200 mW/cm^2^ for 160 s (Fig. 2E). For WA1, Beta, and Gamma variants, 32 J/cm^2^ of 425 nm light at each irradiance significantly decreased SARS-CoV-2 titers by >3 log_10_. Taken together, these results suggest that the total dose of administered 425 nm light drives the inactivation of SARS-CoV-2 rather than the irradiance or administration time.

### 425 nm light inhibits SARS-CoV-2 binding, entry, and replication

**Fig 3:**
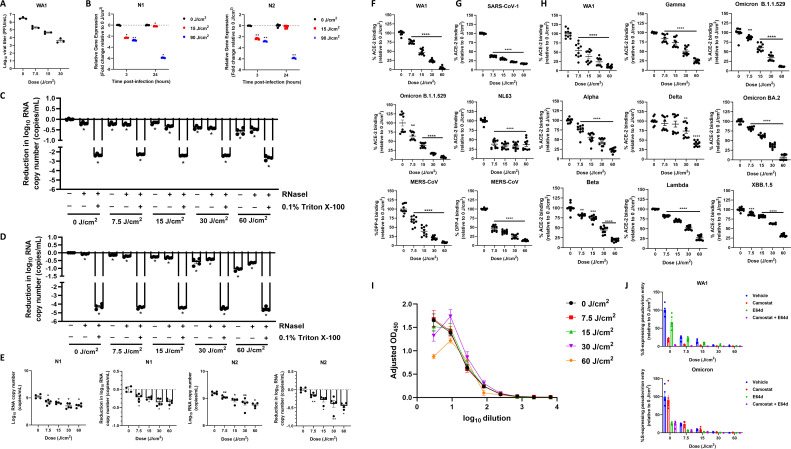
425 nm light inhibits spike binding to ACE-2 and entry to host cells. (**A**) The impact of 425 nm light on active, replication-competent virus replication was assessed via growth curve at 24 h post-infection and plaque assay. Data presented are the mean viral titer (PFU/mL) ± SEM (*n* = 3). (**B**) Vero E6 cells were inoculated with cell-free suspensions of SARS-CoV-2 following illumination with 0, 15, and 90 J/cm^2^ of 425 nm light. At 3 and 24 hpi, total RNA was extracted from inoculated cells for qRT-PCR analysis of N1 and N2 with the CDC RUO assay kit and TaqMan Fast Virus 1-Step Master Mix. Data presented are normalized mean N1 and N2 expression relative to RNase P at 3 and 24 hpi. Heat-inactivated (**C**) and active, replication-competent WA1 (**D**) were illuminated with 425 nm light and incubated with or without RNase I and Triton X-100. RNA copies were determined via qRT-PCR. Data presented are the mean log_10_ RNA copy number reduction relative to 0 J/cm^2^ (–/–) ± SEM (*n* = 4). (**E**) Active, replication-competent SARS-CoV-2 WA1 was illuminated with 425 nm light, and viral RNA copies were determined via qRT-PCR. Data presented are the mean N1 and N2 viral RNA copies ± SEM (*n* = 4–5) and the mean reduction in viral RNA copies relative to 0 J/cm^2^ ± SEM (*n* = 4–5). Recombinant S1 protein (**F**), recombinant S1+S2 proteins (**G**), recombinant spike trimers (**H**), and active, replication-competent SARS-CoV-2 (**I**) were assessed for receptor binding following 425 nm illumination. Data presented are the mean percent binding to ACE-2 or DPP-4 relative to 0 J/cm^2^ ± SEM (*n* = 10 across two independent experiments). (**J**) Pseudovirions expressing WA1 or Omicron spikes were illuminated and evaluated for their entry to susceptible cells via luciferase assay. Data presented are the mean entry relative to 0 J/cm^2^ ± SEM (*n* = 6). Statistical significance was determined via Mann-Whitney rank-sum test and is indicated by * (*P* < 0.05), ** (*P* < 0.01), *** (*P* < 0.001), and ****(*P* < 0.001). For panel B, statistical significance was determined in comparison to the 0 J/cm^2^ group at each timepoint. For panels C and D, statistical significance was determined relative to the 0 J/cm^2^ (–/–) group. For panels E–H, statistical significance was determined relative to the 0 J/cm^2^ group.

We next sought to investigate the impacted viral factors by which 425 nm light inactivates cell-free SARS-CoV-2. The nature of cell-free inactivation suggested that 425 nm light acted on the lipid envelope, genomic RNA, structural proteins, or a combination of these. First, we inoculated Vero E6-TMPRSS2 cells with 425 nm light-illuminated SARS-CoV-2 WA1 and measured viral titer via plaque assay at 24 hpi (Fig. 3A). Illumination reduced SARS-CoV-2 titers in a dose-dependent manner at 24 hpi; however, this dose-dependent decrease correlated with dose-dependent reductions in the initial illumination (Fig. 2), suggesting that illumination did not impair the replication of active, replication-competent virus within the suspension.

Next, we inoculated cells with 425 nm-illuminated SARS-CoV-2 suspensions and measured the viral RNA in cell lysates via qRT-PCR at 3 and 24 hpi (Fig. 3B; Fig. S2). The doses tested were 15 J/cm^2^ for incomplete inactivation and 90 J/cm^2^ to ensure complete inactivation. At 3 hpi, both 15 and 90 J/cm^2^ significantly decreased the viral N gene relative expression by more than twofold compared to the 0 J/cm^2^ control. However, at 24 hpi, the viral N gene relative expression in the 15 J/cm^2^ group had comparable viral N gene relative expression as that of the unilluminated controls. Conversely, the viral N gene relative expression in the 90 J/cm^2^ group significantly decreased by more than sixfold compared to the unilluminated control at 24 hpi. The presence of viral RNA in cell lysates inoculated with fully inactivated virus suggests that 425 nm light-illuminated virus retains the ability to bind, fuse, and/or enter, but not to replicate in, host cells.

Thus, we explored the effect of 425 nm light on the SARS-CoV-2 virion and viral RNA. First, we assessed whether illumination with 425 nm light compromised envelope integrity and exposed viral genomic RNA to degradation by host RNases. To assess this, we conducted an RNase I protection assay with heat-inactivated SARS-CoV-2 WA1 (Fig. 3C; Fig. S3A) and active, replication-competent SARS-CoV-2 WA1 (Fig. 3D; Fig. S3B). Suspensions were illuminated with 425 nm light and incubated with RNase I with and without 0.1% Triton X-100 (+/+ and +/–, respectively); viral RNA copies were quantified using qRT-PCR. In illuminated suspensions of active and inactive virions, 425 nm light reduced the viral N gene RNA copies by a maximum of <0.5 log_10_ and <1 log_10_ viral RNA copies following illumination with 60 J/cm^2^ in heat-inactivated and active suspensions, respectively. Importantly, the addition of RNase I did not lead to a further reduction in viral RNA copies compared to RNase I-untreated samples, suggesting that viral RNA remained protected by the viral envelope. Conversely, 425 nm light strongly reduced the viral N gene RNA copies by >2 log_10_ and >4 log_10_ in heat-inactivated and active suspensions, respectively, when treated with 0.1% Triton X-100 and RNase I following illumination.

Next, we sought to explore the effect of 425 nm light on SARS-CoV-2 viral genomic RNA. To assess how 425 nm light illumination impacted SARS-CoV-2 RNA, we illuminated replication-competent SARS-CoV-2 WA1 and determined the absolute copy number of viral RNA using qRT-PCR (Fig. 3E). In both the N1 and N2 primer/probe sets, viral RNA copy numbers ranged from >9 log_10_ RNA copies to ~8.7 log_10_ RNA copies at 0 and 60 J/cm^2^, respectively. The maximum reduction in viral RNA copy number was <0.5 log_10_ at 60 J/cm^2^ for both primer/probe sets. These results suggested that illumination with 425 nm light does not compromise the viral envelope integrity to expose the viral RNA to environmental degradation but may directly impact viral RNA integrity.

The SARS-CoV-2 spike has served as an antigenic target for multiple prophylactic and therapeutic approaches to prevent and treat infection with SARS-CoV-2 infection and the progression of COVID-19 (14, 30). Thus, we explored whether 425 nm light inhibited spike binding to host receptors via receptor-ligand binding assays with ACE-2 or DPP-4 with recombinant spike proteins (Fig. 3F through H) and active, replication-competent virus (Fig. 3I). First, we utilized recombinant S1 spike domains from SARS-CoV-2 and MERS-CoV (Fig. 3F); recombinant S1+S2 spike proteins from SARS-CoV-1, NL63, and MERS-CoV (Fig. 3G); recombinant spike trimers from SARS-CoV-2 VOCs (Fig. 3H); and active, replication-competent SARS-CoV-2 WA1 (Fig. 3I). Except for the Delta trimer, 425 nm light significantly reduced SARS-CoV-2 spike binding to ACE-2 and MERS-CoV spike binding to DPP-4 in a dose-dependent manner. Despite significant differences at each dose, 425 nm light did not inhibit NL63 S1+S2 protein binding to ACE-2 in a dose-dependent manner; rather, NL63 spike binding to ACE-2 was reduced by approximately 60% at each dose. With the trimeric SARS-CoV-2 spike protein, 425 nm light reduced spike binding to ACE-2 for spike trimers derived from WA1, Alpha, Beta, Gamma, Lambda, Omicron B.1.1.529 and BA.2, and XBB.1.5 in a dose-dependent manner (Fig. 3H). Finally, 425 nm light reduced active, replication-competent SARS-CoV-2 binding to ACE-2 in a dose-dependent manner at the lowest serial dilution, although this effect disappeared at higher dilutions (Fig. 3I). These results suggest that 425 nm light can inhibit the ability of SARS-CoV-2 spike proteins to bind to ACE-2 *in vitro*, although this effect may not extend to active, replication-competent virus.

To determine if 425 nm light impacted SARS-CoV-2 entry to susceptible host cells following ACE-2 binding, we utilized a pseudovirion entry assay (Fig. 3J; Fig. S4). After binding to ACE-2, the SARS-CoV-2 spike requires a proteolytic cleavage step by either TMPRSS2 or cathepsin B/L to fuse with host membranes and enter the cell. To assess if 425 nm light impacted this proteolytic cleavage site, we conducted this assay with inhibitors of TMPRSS2 (camostat mesylate), cathepsin B/L (E64d), both individually and in combination compared to uninhibited controls. As expected, 425 nm light without protease inhibitors reduced the entry of WA1 and Omicron spike-expressing pseudovirions in a dose-dependent manner. The TMPRSS2 inhibitor camostat mesylate and cathepsin E/L inhibitor E64d reduced WA1 spike-expressing and Omicron spike-expressing pseudovirion entry, respectively. However, camostat mesylate and E64d had minimal impact on WA1 and Omicron pseudovirion entry, respectively. In short, these results suggest that 425 nm light does not impact the proteolytic cleavage site on the spike protein.

### Riboflavin augments the inactivation of SARS-CoV-2 by 425 nm light

**Fig 4:**
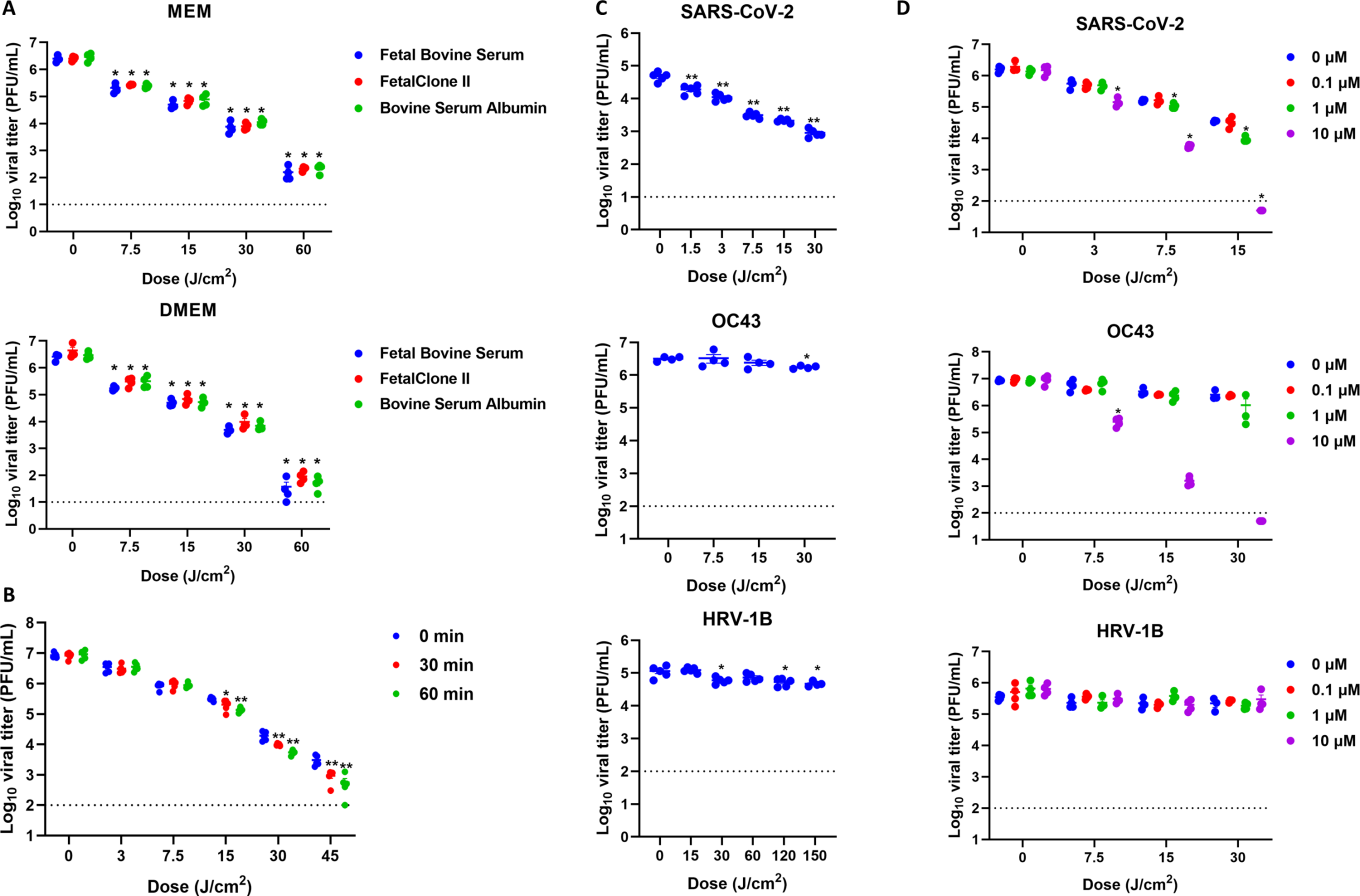
Inactivation of SARS-CoV-2 by 425 nm light is augmented by photosensitizers in cell culture media. (**A**) SARS-CoV-2 WA1 was propagated in basal media composed of MEM and DMEM with different serum supplementation (FBS, BSA, or FetalCloneII). Cell-free suspensions of SARS-CoV-2 (*n* = 4) were illuminated with 425 nm light and enumerated via plaque assay. (**B**) Cell culture media were illuminated with 425 nm light and used as basal media for SARS-CoV-2 stock generation. SARS-CoV-2 WA1 was diluted in corresponding media and illuminated with 425 nm light followed by plaque assay. Data presented are mean viral titers (PFU/mL) ± SEM (*n* = 5). (**C**) SARS-CoV-2 WA1, OC43, and HRV-1B were diluted in artificial saliva (BioChemaZone BZ108) and illuminated with 425 nm light followed by plaque assay. Data presented are mean viral titers (PFU/mL) ± SEM (*n* = 4–5). (**D**) SARS-CoV-2 NY, OC43, and HRV-1B were diluted in artificial saliva (BioChemaZone BZ108) supplemented with riboflavin and illuminated with 425 nm light followed by plaque assay. Data presented are mean viral titers (PFU/mL) ± SEM (*n* = 4). Statistical significance was determined via Mann-Whitney rank-sum test and is indicated by * (*P* < 0.05) and ** (*P* < 0.01). For panel A, statistical significance was determined relative to the corresponding 0 J/cm^2^ group in each media formulation. For panel B, statistical significance was determined relative to the 0 min group at each illuminated dose. For panel C, statistical significance was determined relative to the 0 J/cm^2^ group. For panel D, statistical significance was determined relative to the 0 µM group at the corresponding illumination dose.

Illumination with 425 nm light can occur in the presence or absence of photosensitizers like riboflavin. The diluent used in the above studies consisted of cell culture media supplemented with serum that contained different photosensitizing compounds at different concentrations. For instance, DMEM contains approximately four times higher concentrations of amino acids and vitamins than MEM. To assess how medium composition impacted 425 nm light inactivation of SARS-CoV-2, we propagated SARS-CoV-2 WA1 stocks in MEM or DMEM supplemented with FBS, FetalCloneII, and bovine serum albumin (BSA). Stocks were diluted in the same medium and illuminated with 425 nm light followed by plaque assay (Fig. 4A). Illumination significantly reduced SARS-CoV-2 titers in a comparable dose-dependent manner regardless of basal medium formulation or serum supplementation. To further assess how culture medium impacted 425 nm light inactivation, we illuminated DMEM supplemented with FBS for 30 min (90 J/cm^2^) or 60 min (180 J/cm^2^); after illumination, SARS-CoV-2 stocks were generated in this medium and illuminated with 425 nm light. In both unilluminated and illuminated media, 425 nm light significantly reduced SARS-CoV-2 titers in a dose-dependent manner (Fig. 4B). Interestingly, we observed a further trend toward reduction in SARS-CoV-2 in illuminated media at 30 and 45 J/cm^2^ relative to unilluminated media. These results suggest that 425 nm light inactivates SARS-CoV-2 regardless of the standard basal media composition, although prior photoactivation of the basal media can augment inactivation.

Previous studies have indicated that 405 nm light can inactivate SARS-CoV-2 diluted in photosensitizer-free PBS (24). In the present study, we evaluated whether dilution in artificial saliva impacted the inactivation of SARS-CoV-2 WA1, HRV-1B, and OC43 by 425 nm light (Fig. 4C). In artificial saliva, 425 nm light significantly inactivated SARS-CoV-2 in a dose-dependent manner; however, these reductions were reduced compared to the inactivation in diluent (Fig. 2A). Specifically, 425 nm light reduced SARS-CoV-2 titers by >1 log_10_ at 7.5 J/cm^2^ but did not achieve 2 log_10_ reduction at 15 or 30 J/cm^2^ in artificial saliva. A similar trend was observed with OC43; 30 J/cm^2^ of 425 nm light significantly reduced viral titer by <1 log_10_ in saliva compared to >1 log_10_ in culture medium (Fig. 2C). For HRV-1B, 425 nm light significantly reduced viral titers at 30, 120, and 150 J/cm^2^; these differences were comparable to those previously observed in culture medium (23). These results suggest that culture medium and the concentration of photosensitizers impact 425 nm light inactivation of SARS-CoV-2.

Finally, we wanted to assess the impact of riboflavin on 425 nm light inactivation of SARS-CoV-2. Riboflavin is a known photosensitizer that is present in cell culture media (31). Riboflavin is present at a concentration of approximately 0.25 and 1 µM in MEM and DMEM, respectively. Thus, we diluted SARS-CoV-2, OC43, and HRV-1B in artificial saliva supplemented with 0, 0.1, 1, or 10 µM riboflavin prior to illumination with 425 nm light (Fig. 4D). Riboflavin supplementation increased inactivation kinetics in a dose-dependent manner for SARS-CoV-2 and OC43 but not for HRV-1B. Specifically, 1 µM riboflavin significantly increased inactivation at 7.5 and 15 J/cm^2^, and 10 µM increased inactivation at each dose tested relative to the 0 µM group at the corresponding dose. At 15 J/cm^2^, 10 µM riboflavin reduced SARS-CoV-2 titers by >2 log_10_ compared to the 0 J/cm^2^ group. A similar trend was observed with OC43; 425 nm illumination and supplementation with 10 µM riboflavin resulted in reductions >1 log_10_, >3 log_10_, and >4 log_10_ at 7.5 , 15, and 30 J/cm^2^, respectively, compared to the corresponding 0 µM group. Conversely, riboflavin supplementation did not impact 425 nm light inactivation of HRV-1B. These data indicate that riboflavin is a photosensitizer for 425 nm light and that riboflavin supplementation augments 425 nm light inactivation of SARS-CoV-2 and OC43.

### 425 nm light reduces SARS-CoV-2 Beta, Delta, and Omicron titers in ALI HAE

**Fig 5:**
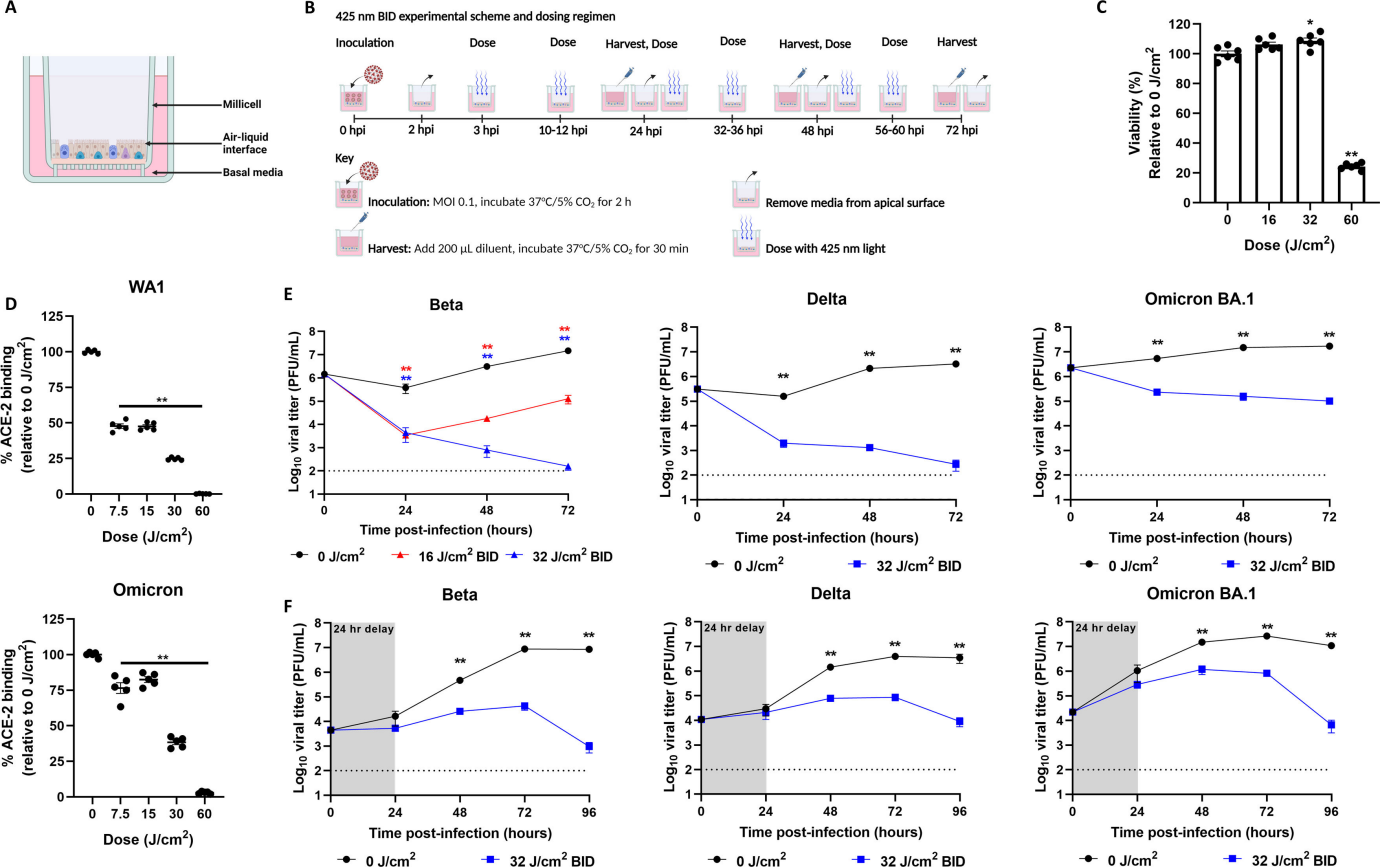
425 nm light reduces SARS-CoV-2 Beta, Delta, and Omicron titers at non-cytotoxic doses in ALI HAE. Well-differentiated human large airway epithelial cells (**A**) were illuminated twice daily (BID) with 16 or 32 J/cm^2^ of 425 nm light (**B**). At 72 hpi (six total doses), cell viability was determined via 3-(4,5-dimethylthiazol-2-yl)-2,5-diphenyltetrazolium bromide (MTT) assay (**C**). Data presented are the mean viability ± SEM relative to 0 J/cm^2^ (*n* = 6). (**D**) Plates coated with synthetic human ACE-2 were illuminated with 425 nm light and inoculated with recombinant spike trimers derived from WA1 and Omicron. Data presented are the mean percent binding to ACE-2 relative to 0 J/cm^2^ ± SEM (*n* = 5). (**E, F**) ALI HAE cells were infected with SARS-CoV-2 Beta, Delta, or Omicron variants, treated, and illuminated twice daily for 3 days with 32 J/cm^2^ of 425 nm light starting at 3 hpi (MOI 0.1 [**E**]) or 24 hpi (MOI 0.001 [**F**]). Apical rinses were collected daily and enumerated via plaque assay. Data presented are the mean viral titer (PFU/mL) ± SEM (*n* = 5–6). Statistical significance was determined with the Mann-Whitney rank-sum test and is indicated by * (*P* < 0.05) and ** (*P* < 0.01). For panels C and D, statistical significance was determined relative to the 0 J/cm^2^ group. For panels E and F, statistical significance was determined relative to the 0 J/cm^2^ group at the corresponding timepoint.

We previously demonstrated that a twice-daily 425 nm light dosing regimen could reduce SARS-CoV-2 WA1 titers in well-differentiated ALI HAE cell cultures derived from tracheobronchial primary cells (Fig. 5A) (23). As SARS-CoV-2 VOCs have demonstrated increased replication *in vitro* relative to WA1 (32–35), we assessed whether 16 or 32 J/cm^2^ of 425 nm light in a twice-daily (BID, separated by at least 8 h) dosing regimen starting at 3 hpi would retain antiviral capability against the SARS-CoV-2 Beta VOC in the same model (Fig. 5B). First, we observed that the 16 and 32 J/cm^2^ BID dosing schedules did not reduce the viability of time-matched, uninfected airway epithelial cells at 72 hpi compared to the 0 J/cm^2^ control following six total doses (Fig. 5C), indicating that these doses are not cytotoxic to this model. However, increasing the dose to 60 J/cm^2^ BID significantly reduced viability by 76% relative to the 0 J/cm^2^ control, suggesting that doses less than 60 J/cm^2^ should serve as the basis for a non-irritating antiviral (Fig. 5C).

Next, we examined the impact of illumination on ACE-2 by modifying the above receptor-ligand binding assay. In this assay, we illuminated plates coated with ACE-2 prior to the addition of unilluminated WA1 and Omicron spike trimers. Interestingly, 425 nm illumination reduced spike binding to ACE-2 but not in a dose-dependent manner (Fig. 5D). For WA1 and Omicron, 7.5 and 15 J/cm^2^ reduced the binding by >50% and >10%, respectively, while 60 J/cm^2^ abrogated spike binding to ACE-2 for both variants. Based on these results, we focused on the 16 and 32 J/cm^2^ BID doses to determine if 425 nm light could inhibit SARS-CoV-2 Beta variant titers in the ALI HAE model.

In ALI HAE infected with the SARS-CoV-2 Beta variant (MOI 0.1), both the 16 and 32 J/cm^2^ BID dosing regimens significantly reduced viral titers at each timepoint relative to the 0 J/cm^2^ control (Fig. 5E). Specifically, the 16 and 32 J/cm^2^ BID dosing regimen reduced by 2.2 log_10_ and 5.1 log_10_ at 72 hpi. Despite comparable viral titers at 24 hpi, the 32 J/cm^2^ BID dosing regimen further reduced viral titers by 1.6 log_10_ at 48 hpi and 2.8 log_10_ at 72 hpi compared to the 16 J/cm^2^ BID dosing regimen. Thus, we further explored the 32 J/cm^2^ BID dosing regimen against the Delta and Omicron variants. The 32 J/cm^2^ BID regimen significantly reduced Delta and Omicron titers by 4.2 log_10_ and 2.2 log_10_ at 72 hpi compared to the 0 J/cm^2^ control group, respectively (Fig. 5E). Taken together, these results indicate that a BID dosing regimen of 32 J/cm^2^ of 425 nm light can reduce SARS-CoV-2 VOC viral titers in ALI HAE.

To investigate if the 425 nm 32 J/cm^2^ BID dosing regimen retained antiviral efficacy in more established infections, we infected the airway epithelia model with the Beta, Delta, or Omicron variants (MOI 0.001) and delayed the administration of the first dose to 24 hpi (Fig. 5F). For each variant, viral titers did not differ significantly at 24 hpi (prior to the administration of the first dose), but 425 nm light significantly reduced titers at each timepoint afterward. Specifically, 425 nm light reduced the Beta, Delta, and Omicron variants by 4.2 log_10_, 2.7 log_10_, and 3.6 log_10_, respectively, at 96 hpi relative to the 0 J/cm^2^ control group (Fig. 5F). These results indicate that the 425 nm 32 J/cm^2^ BID dosing regimen can reduce viral titers following early (first dose at 3 hpi) or delayed (first dose at 24 hpi) dose administrations in human airway epithelial models of SARS-CoV-2 infections.

### The RD-X19 reduces spike-ACE-2 binding, inactivates SARS-CoV-2, and reduces SARS-CoV-2 titers in ALI HAE

**Fig 6:**
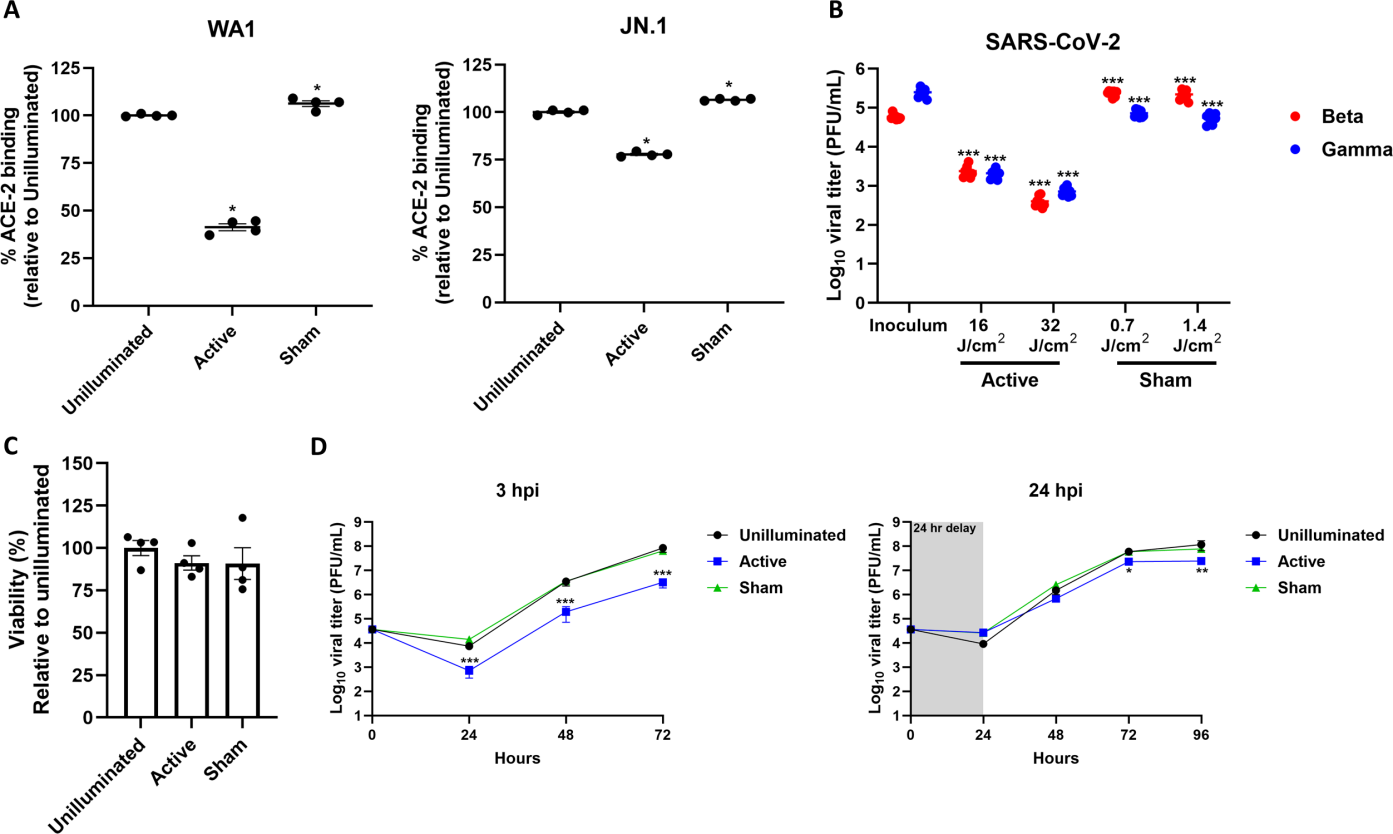
The RD-X19 reduces spike binding to ACE-2, inactivates SARS-CoV-2, and reduces viral titers at non-cytotoxic doses. (**A**) Recombinant spike trimers derived from WA1 and Omicron JN.1 were assessed for receptor binding following illumination with the active RD-X19 or sham. Data presented are the mean percent binding to ACE-2 relative to unilluminated ± SEM (*n* = 4). (**B**) SARS-CoV-2 Beta and Gamma were diluted in cell culture media and illuminated with the active RD-X19 or sham. Data presented are the mean viral titer (PFU/mL) ± SEM (*n* = 8). (**C**) ALI HAE cell cultures were illuminated twice daily (BID) with the Active RD-X19 or Sham for 3 days (six total doses). Cell viability was determined via MTT assay. Data presented are the mean viability ± SEM relative to unilluminated (*n* = 4). (**D**) ALI HAE cell cultures were infected with SARS-CoV-2 WA1 illuminated twice daily for 3 days with the active RD-X19 or sham starting at 3 or 24 hpi. Apical rinses were collected daily and enumerated via plaque assay. Data presented are the mean viral titer (PFU/mL) ± SEM (*n* = 10). Statistical significance was determined with the Mann-Whitney rank-sum test and is indicated by * (*P* < 0.05), ** (*P* < 0.01), and *** (*P* < 0.001). For panel A, statistical significance was determined relative to the 0 J/cm^2^ group. For panel B, statistical significance was determined relative to the inoculum. For panel D, statistical significance was determined relative to the unilluminated group at the corresponding timepoint.

While the BLU demonstrated utility as a high-throughput, preclinical platform, its setup may not replicate the use of a medical device emitting 425 nm light to treat SARS-CoV-2 in clinical studies. Thus, we developed an *in vitro* test platform to evaluate the antiviral capability of the RD-X19, a clinically studied medical device that emits a nominal dose of 32 J/cm^2^ of 425 nm light to the oropharynx to treat upper respiratory viral infections. For comparison, we used a sham device that emits 470 nm light at a fluence more than 20-fold lower than that of the active device. Using this platform, we evaluated how the RD-X19 inhibited spike binding to ACE-2, inactivated SARS-CoV-2 VOCs, and reduced SARS-CoV-2 titers in ALI HAE models (Fig. 6). Using recombinant WA1- and JN.1-derived stabilized spike trimers, we observed that 425 nm light reduced spike binding to recombinant ACE-2 (Fig. 6A). RD-X19 active illumination significantly reduced WA1 and JN.1 spike binding to ACE-2 by >50% and ~25%, respectively, while sham illumination had no impact on spike binding to ACE-2. Furthermore, the RD-X19 active device reduced SARS-CoV-2 Beta titers by >1 log_10_ at 16 J/cm^2^ and by >2 log_10_ at 32 J/cm^2^, and SARS-CoV-2 Gamma titers by >2 log_10_ at both doses, while the sham devices were comparable to the starting inoculum (Fig. 6B). Finally, neither the RD-X19 active nor the sham devices induced minimal cytotoxicity in ALI HAE following twice-daily dosing for 3 days (Fig. 6C). In SARS-CoV-2 WA1-infected ALI HAE, twice-daily dosing initiated at 3 hpi with the RD-X19 active device significantly reduced viral titers at each timepoint, while the sham device did not (Fig. 6D). Specifically, the RD-X19 active device reduced SARS-CoV-2 titers by >1 log_10_ at 48 and 72 hpi relative to the unilluminated control. Additionally, initiating the dosing at 24 hpi significantly reduced the viral titer relative to unilluminated controls, although the magnitude of reduction was less than when initiated at 3 hpi. Specifically, the peak viral titer reduction in this dosing regimen was approximately 0.5 log_10_ at 72 and 96 hpi. In both dosing regimens, the sham device titers mirrored those observed in the unilluminated control. Taken together, these results indicate that, like the BLU, the RD-X19 medical device exhibits *in vitro* antiviral effects that are consistent with its clinical results, although the magnitude of significantly reduced viral titer is smaller than was observed with the BLU.

## DISCUSSION

The rapid development of vaccines and therapeutics combined with supportive public health measures reduced the worldwide health and economic burdens associated with COVID-19. As public health measures have eased, vaccination rates decline, viral evolution drives the emergence of therapeutic-resistant variants, and SARS-CoV-2 becomes an endemic respiratory pathogen, it is plausible that more variants will arise. Thus, the development of a pan-variant therapeutic to complement existing therapeutics is needed to minimize the health and economic impacts of SARS-CoV-2. Previous studies have indicated that visible light reduces the titers of SARS-CoV-2 and other betacoronaviruses in cell-free suspensions (23–25, 36). Furthermore, non-cytotoxic doses of visible light reduce SARS-CoV-2 titers in immortalized cell cultures (23, 26) and in well-differentiated models of the human buccal epithelium (27) and human large airway epithelium (23). Since these reports, SARS-CoV-2 has mutated, and five VOCs have been identified by the WHO. In this study, we explore the potential for visible light to serve as a pan-variant countermeasure; we demonstrate that 425 nm light inactivates representative isolates of each of the five SARS-CoV-2 VOCs in cell-free suspensions by reducing viral entry. Furthermore, we show that a twice-daily dosing regimen with 425 nm light reduces SARS-CoV-2 VOC titers in ALI HAE models at multiple stages of infection. Finally, we demonstrate the antiviral efficacy of the RD-X19, a medical device emitting 425 nm light.

The five representative SARS-CoV-2 VOCs utilized in this study contain mutations associated with both immune evasion and increased transmissibility. For example, immune evasion mutations include spike L18F (Beta, Gamma) (37), spike Δ157-158 (Delta) (38), spike L452R (Delta) (39), spike E484K (Beta, Gamma) (40), spike E484A (Omicron) (41, 42), and spike N501Y (Alpha, Beta, Gamma, Omicron) (37, 39, 41–44). Additionally, the VOC panel incorporated mutations associated with increased transmissibility including spike L452R (Delta) (39), spike N501Y (Alpha, Beta, Gamma, Omicron) (45), spike D614G (Alpha, Beta, Gamma, Delta, Omicron) (32–34), spike P681R (Delta) (44, 46), nucleocapsid R203K (Alpha, Gamma, Delta) (47, 48), and nucleocapsid G204R (Alpha, Gamma, Delta) (47, 48). Despite these constellations of mutations, SARS-CoV-2 VOCs remain as equally susceptible as WA1 to 425 nm light inactivation (23). Furthermore, bamlanivimab effectively neutralized WA1 and the Alpha VOC but did not neutralize the Beta, Gamma, or Delta VOCs at the concentrations evaluated in this study (20, 49) and did not neutralize the Omicron variants (19, 20, 29). Taken together, these results indicate that antibody escape mutations and/or transmission mutations do not negatively impact the potency of 425 nm light inactivation of SARS-CoV-2 VOCs. These results provide further evidence that 425 nm visible light has the potential to serve as a pan-variant SARS-CoV-2 antiviral.

Given the pan-variant inactivation of cell-free SARS-CoV-2 by 425 nm light, we considered viral factors impacted by 425 nm light within this system. We focused on the inhibition of viral binding and entry to the host cell. Viral inactivation with ultraviolet light occurs via fracturing the viral genome, mutagenesis of the genome with pyrimidine dimers, and/or cross-linking or damage to viral proteins (50). Previous work has shown that visible light is a weak inducer of mutagenesis in RNA (51) and likely inactivates RNA viruses via reactive oxygen species (ROS)-induced RNA–RNA or RNA–protein cross-linking, modifications of the viral envelope and/or structural glycoproteins, RNA genome degradation, or a combination of these (52). This non-specific action by ROS contributes to the pan-variant inactivation by 425 nm light. Additionally, non-enveloped viruses require higher doses for inactivation than enveloped viruses, and DNA viruses are more resistant to inactivation than RNA viruses (reviewed by Hessling et al. [53]). Furthermore, within the context of enveloped viruses, visible light exhibited differential inactivation kinetics for SARS-CoV-1, SARS-CoV-2, MERS-CoV, respiratory syncytial virus, influenza A virus, OC43, and NL63 (22–24, 36, 53). Taken together, these findings suggest that 425 nm light functions primarily through action on the lipid envelope followed by structural glycoproteins and nucleic acids.

A previous study indicated that 420 nm light does not result in spike denaturation but did not assess whether spike binding to ACE-2 was impacted (36). The present study suggests that 425 nm light inactivates SARS-CoV-2 by preventing viral binding and entry to the host cell. We reached this conclusion based on multiple findings. First, 425 nm light had reduced or minimal activity against multiple RNA viruses, including the CoVs NL63 and OC43, suggesting that 425 nm light does not act on the CoV RNA genome. Second, 425 nm light inhibited SARS-CoV-2, but not NL63, spike binding to ACE-2, although this inhibition was not complete. Third, the presence of viral RNA in cell lysate suggests that illuminated suspensions contained an intact virion that could bind but could not fuse or enter host cells, likely resulting from ROS-induced lipid peroxidation of the viral envelope. Notably, these results suggest that there is not a single mechanism by which 425 nm light inactivates SARS-CoV-2; rather, the non-specific interactions of ROS with viral components allow multiple avenues for the broad-spectrum inactivation of SARS-CoV-2 variants.

Photomedicine functions via photobiomodulation (PBM) or photodynamic therapy (PDT). Whereas PBM involves the interaction of visible light with endogenous pathways in a biological system, PDT requires exogenous photosensitizers to exert its effects through the generation of ROS (54). Previous studies have indicated that both PBM and PDT can inactivate human CoVs (24, 36, 55, 56). The present study evaluated inactivation in cell culture media and artificial saliva; cell culture media and unpurified viral stocks contain known visible light photosensitizers (e.g., riboflavin, porphyrins) that can impact viral inactivation by visible light (31, 53). For example, fluorescent illumination of exogenous porphyrins inactivated SARS-CoV-2 via generation of singlet oxygen that was reduced by sodium azide scavenging (56). Previous works demonstrated that the dilution of SARS-CoV-2 within artificial saliva or PBS did not result in loss of inactivation potency relative to dilution in cell culture media (22, 24, 25). For instance, visible light illumination of viral suspensions in PBS without photosensitizers reduced cell-free SARS-CoV-2 titers by 2.31 log_10_ at 17.28 J/cm^2^ (24), which is comparable to the titer reductions observed at the 15 J/cm^2^ dose for each variant tested in media in the present study. Additionally, we noted that basal medium or serum supplementation did not alter viral inactivation kinetics (Fig. 4A). However, we did observe that dilution in artificial saliva did reduce inactivation in our system (Fig. 4C). One plausible explanation is that dilution in artificial saliva diluted exogenous photosensitizers, such as riboflavin and porphyrins. Indeed, supplementation with riboflavin restored viral inactivation kinetics comparable to those in cell culture medium (Fig. 4D). Similarly, increased pre-illumination of media resulted in augmented viral inactivation potentially owing to increased excitation of riboflavin and porphyrins (Fig. 4B). Another plausible explanation is that porphyrins or flavins bind to or integrate into the viral envelope during viral replication and egress (57–59). Such an interaction would provide an endogenous photosensitizer as observed in bacteria; subsequent illumination of endogenous porphyrins with 400–470 nm light has been referred to as antimicrobial blue light (aBL) (60, 61). During replication, CoV structural proteins translocate to the endoplasmic reticulum (ER), and virion assembly occurs during trafficking from the ER to the Golgi via the ER–Golgi intermediate complex (62); porphyrins have been identified within the ER and Golgi *in vitro* (63). Taken together, these results indicate that, within our system, 425 nm light can function through PBM, but its effects are augmented by PDT via exogenous photosensitizers like riboflavin.

The antiviral capability of visible light is an area of burgeoning interest. Studies have demonstrated that dosing virus-infected cells or tissues with light has the capacity to reduce infectious titers. For instance, PBM with blue light reduced SARS-CoV-2 titers in cell cultures (23, 26, 64, 65). Similar effects have also been observed in ALI HAE or well-differentiated buccal epithelium that more accurately reflect translational efficacy and safety than immortalized, two-dimensional cell cultures (23, 27, 66–68). ALI HAE cell cultures display similar physiologic features as human respiratory epithelium *in vivo*, including cilia and mucus production (28), and have shown promising translational accuracy to the clinic (6, 7, 11, 12, 23, 28, 69, 70), including the anti-SARS-CoV-2 therapeutics PF-332 (71), β-D-N^4^-hydroxycytidine (69), and remdesivir (70). PF-332 (71), β-D-N^4^-hydroxycytidine (69), and remdesivir (70) reduced SARS-CoV-2 titers by ~3 log_10_ at 48 hpi. In comparison, 32 J/cm^2^ of 425 nm light delivered twice daily reduced WA1, Beta, and Delta titers by ~3 log_10_, and Omicron titers by ~2 log_10_ at comparable timepoints when dosing began at 3 or 24 hpi (23). That is, 425 nm light reduced viral titers to a similar magnitude as that of PF-332, β-D-N^4^-hydroxycytidine, and remdesivir despite the differences in dosing regimens (69–71). Specifically, discontinuous administration of 425 nm light provided comparable results to continuously administered PF-332, β-D-N^4^-hydroxycytidine, and remdesivir. These benchtop findings contributed to the development of the RD-X19, a medical device that emits 425 nm light targeted to the oropharynx to prevent upper respiratory infections from progressing to the lower respiratory tract. In a benchtop study with the RD-X19, the device reduced SARS-CoV-2 titers in ALI HAE cultures at non-cytotoxic doses. The strongest effects were observed when administration began earlier in the infection process with both the RD-X19 and the BLU, although the magnitude of reduction was superior in the BLU; this difference is a result of the increased number of LEDs in the BLU compared to the RD-X19. However, it is worth knowing that in a 31-subject clinical trial, the RD-X19 reduced SARS-CoV-2 viral load and time to sustained symptom resolution in subjects with mild-to-moderate COVID-19 (27). Thus, these results are encouraging for the further development of 425 nm light as an antiviral against SARS-CoV-2.

The present study has multiple limitations that warrant further investigation. First, RNA damage from 425 nm was not assessed in the studies conducted. While we can conclude there was limited RNA damage within the qRT-PCR amplified region, this does not necessarily reflect the RNA damage or fragmentation outside of the amplified region. Second, the use of unpurified viral stocks included the presence of unpackaged viral RNA that increases the detected viral RNA by qRT-PCR and may not accurately represent the packaged viral RNA. Third, in the current study, we focused on the impacts of 425 nm light on the CoV spike protein. Further work is needed to assess the impacts on other CoV structural proteins, including envelope (E), membrane (M), and nucleocapsid (N). Fourth, while we investigated the role of riboflavin in cell-free inactivation, we did not investigate porphyrins, temperature, or specific ROS and their roles in the inactivation of SARS-CoV-2. In particular, the wavelength-dependent inactivation of SARS-CoV-2 suggests that porphyrins are a prominent contributor to the observed effects (56). Future experiments using specific ROS quenchers (e.g., sodium azide, superoxide dismutase, etc.) or oxygen depletion will further elucidate the responsible flavin, porphyrin, and ROS that result in SARS-CoV-2 inactivation by 425 nm light. Finally, we did not evaluate the mechanisms by which 425 nm light reduces viral titers in ALI HAE models. Specifically, we did not identify whether 425 nm light functions strictly through extracellular inactivation, induces sufficient intracellular ROS, or modulates ACE-2 expression to inhibit viral replication, packaging, or egress; this is an area of ongoing study.

In conclusion, our work shows that 425 nm light inactivates extracellular SARS-CoV-2 in a dose-dependent manner, primarily through inhibition of cellular entry and inhibition of viral binding to the ACE-2 receptor. Furthermore, we show that this approach reduces viral titers in translational models of the human airway following infection with SARS-CoV-2 VOCs at doses that are not hazardous in preclinical models (22). The RD-X19 has been studied in four clinical trials as a treatment for mild-to-moderate COVID-19 (NCT04557826, NCT04662671, NCT04966013, NCT05817045) and has been shown to reduce the time to sustained symptom resolution and viral titers in saliva (27). The preclinical studies described here have provided further evidence to support the clinical development of 425 nm light as a potential antiviral against SARS-CoV-2 VOCs. Our work suggests that photomedicine utilizing 425 nm visible light could serve as the foundation for a new broadly applicable, pan-variant treatment modality for SARS-CoV-2 infection and COVID-19.

## SUPPLEMENTAL MATERIAL

Fig. S1 msphere.00230-25-s0001.TIF Light and monoclonal antibody PRNT.

Fig. S2 msphere.00230-25-s0002.TIF Cell entry raw data.

Fig. S3 msphere.00230-25-s0003.TIF Viral envelope RNase activity raw data.

Fig. S4 msphere.00230-25-s0004.TIF Pseudovirion entry raw data.

Legends msphere.00230-25-s0005.docx Supplemental figure legends.

ASM does not own the copyrights to Supplemental Material that may be linked to, or accessed through, an article. The authors have granted ASM a non-exclusive, world-wide license to publish the Supplemental Material files. Please contact the corresponding author directly for reuse.
