# Supplementary figures and images for: The pan-variant potential of light: 425 nm light inactivates SARS-CoV-2 variants of concern and non-cytotoxic doses reduce viral titers in human airway epithelial cells
Source: mSphere. 2025 May 28;10(6):e00230-25. doi: 10.1128/msphere.00230-25 (PMC12188739; doi:10.1128/msphere.00230-25)

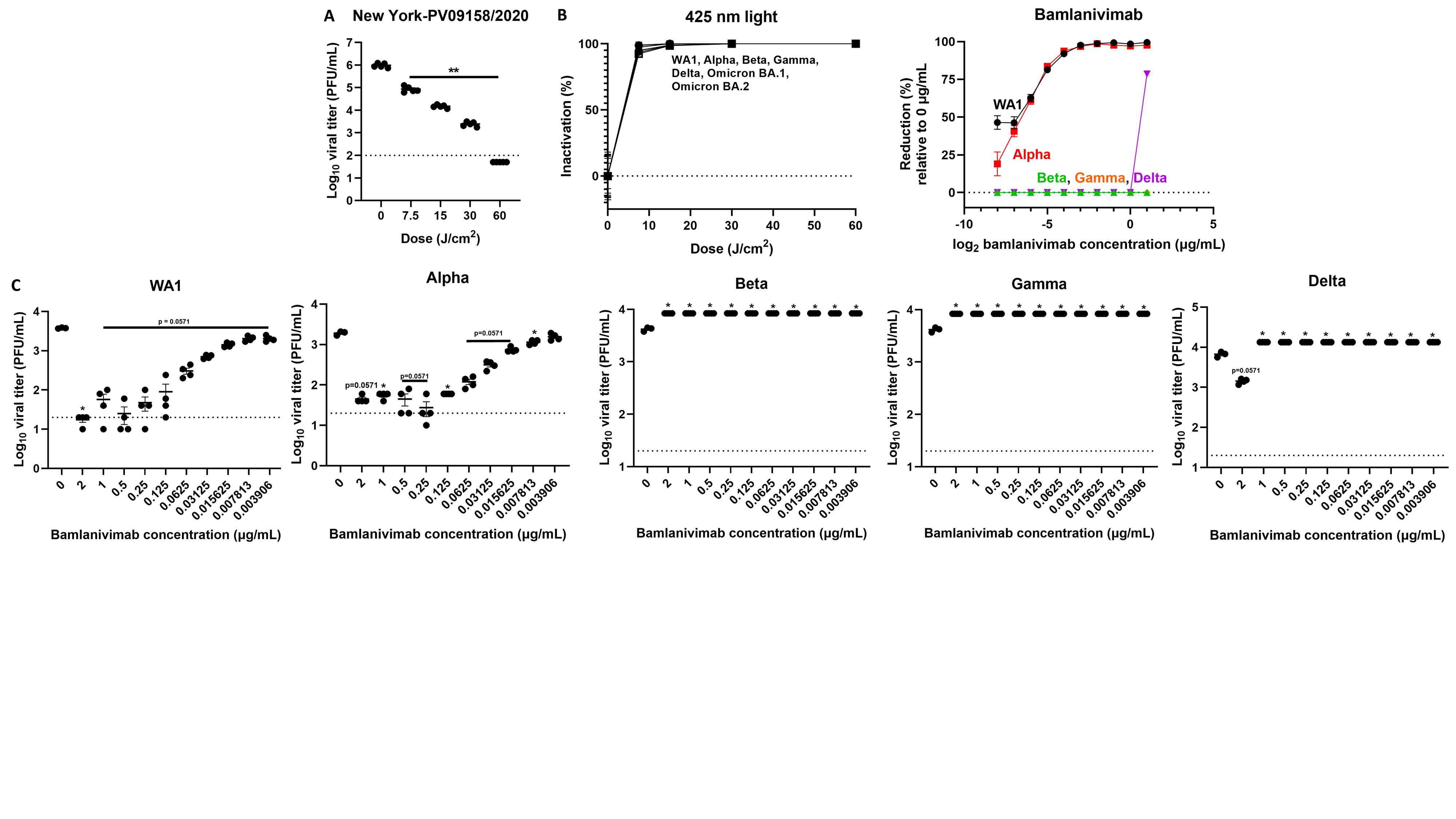

Supplement: Fig. S1 — Light and monoclonal antibody PRNT. [file msphere.00230-25-s0001.tif]

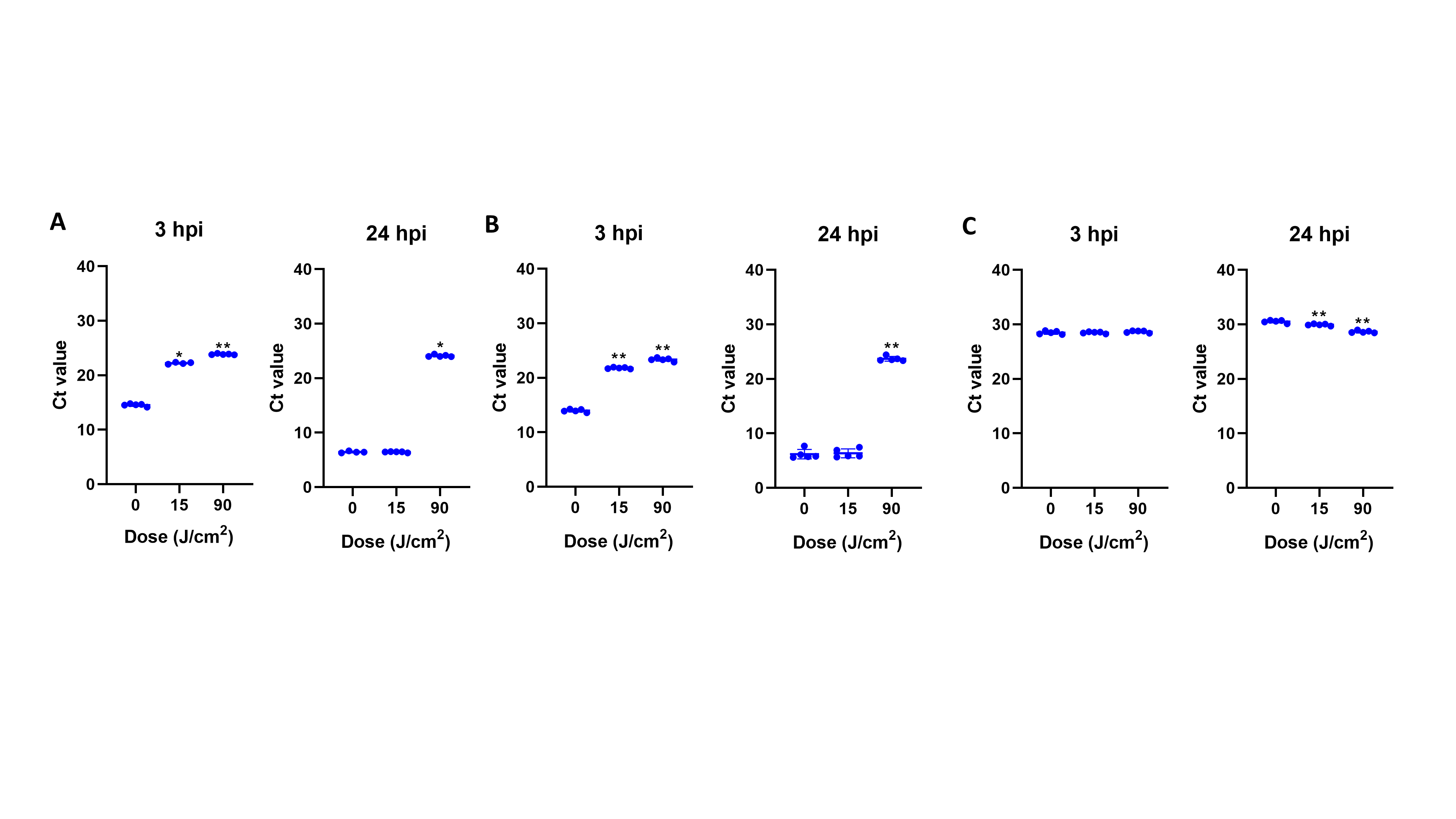

Supplement: Fig. S2 — Cell entry raw data. [file msphere.00230-25-s0002.tif]

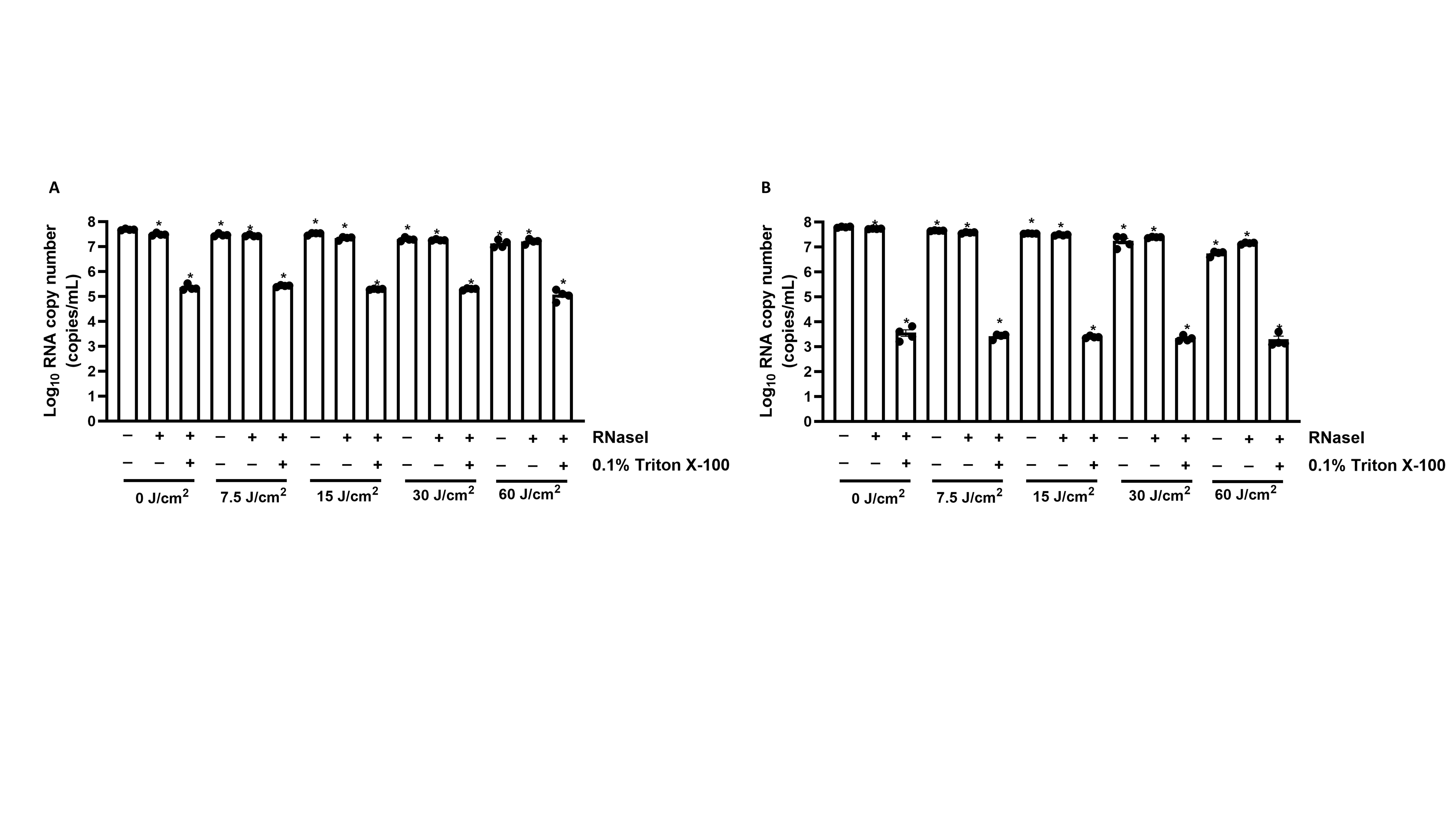

Supplement: Fig. S3 — Viral envelope RNase activity raw data. [file msphere.00230-25-s0003.tif]

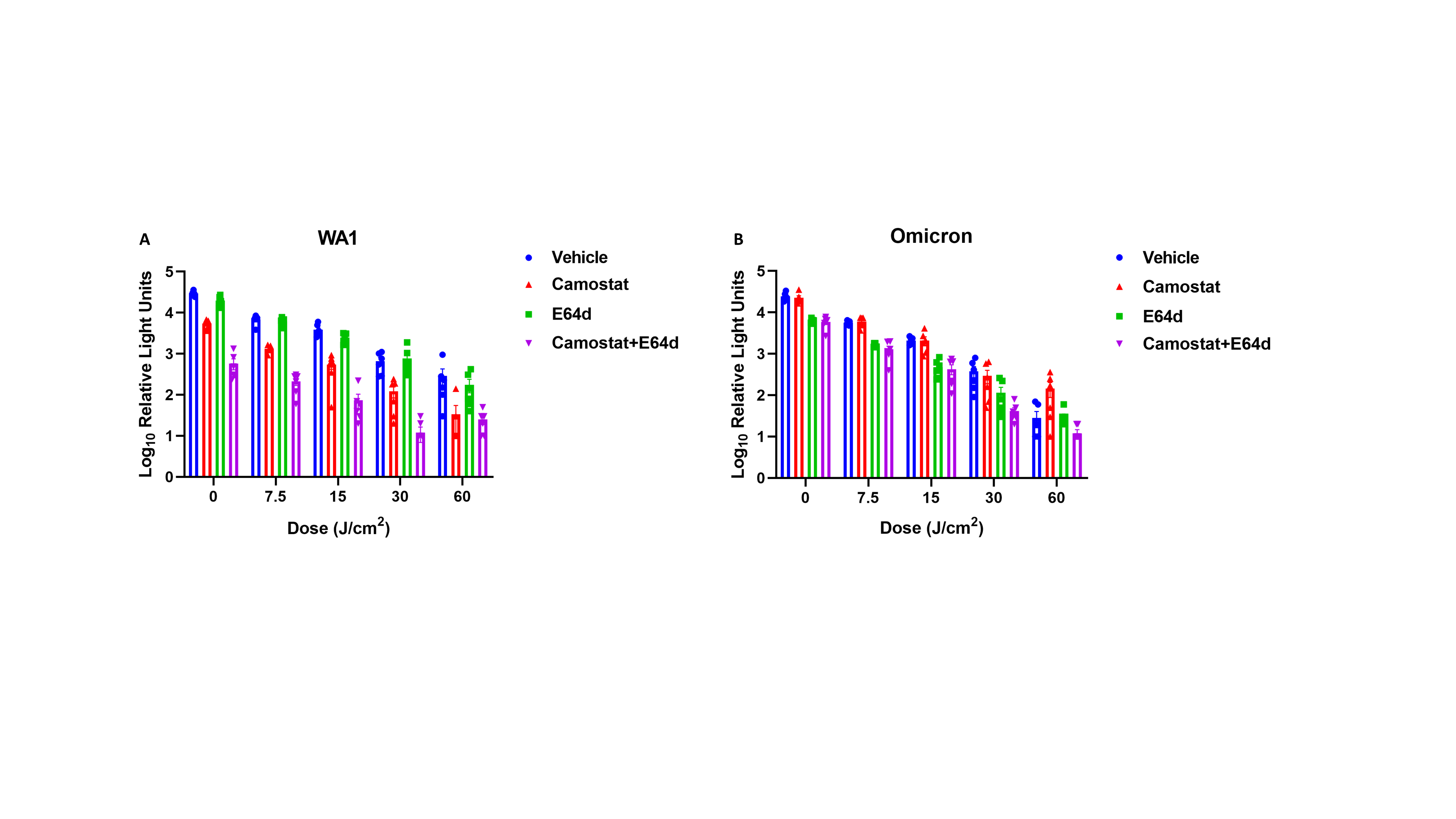

Supplement: Fig. S4 — Pseudovirion entry raw data. [file msphere.00230-25-s0004.tif]
