# Supplementary material for: The pan-variant potential of light: 425 nm light inactivates SARS-CoV-2 variants of concern and non-cytotoxic doses reduce viral titers in human airway epithelial cells
Source: mSphere. 2025 May 28;10(6):e00230-25. doi: 10.1128/msphere.00230-25 (PMC12188739; doi:10.1128/msphere.00230-25)
Supplement: Legends — Supplemental figure legends. [file msphere.00230-25-s0005.docx]

**Supporting information**

**Figure S1. SARS-CoV-2 variants Beta, Gamma, and Delta evade neutralization by the monoclonal antibody therapeutic bamlanivimab.** The capability of 425 nm light to inactivate SARS-CoV-2 NY was evaluated via the PRNT assay. Cell-free stocks of SARS-CoV-2 NY were illuminated with dose range of 425 nm light (A). Data presented are mean viral titers (PFU/ml) +/- SEM (n = 5). The neutralization capability of 425 nm light was compared to bamlanivimab (B) with cell-free SARS-CoV-2 variants (WA1, Alpha, Beta, Delta, and Gamma) via the plaque reduction neutralization test. Cell-free SARS-CoV-2 variants were incubated with varying concentrations of bamlanivimab and viral titers were enumerated via plaque assay (C). Data presented are the mean percent inactivation by 425 nm light +/- SEM (n = 5-6) and mean percent neutralization +/- SEM (n = 4) across bamlanivimab concentrations (ng/mL) relative to 0 J/cm^2^ or 0 ng/mL, respectively. For A and C, statistical significance was determined in comparison to the 0 J/cm^2^.

**Figure S2. 425 nm light inhibits cell-free SARS-CoV-2 entry.** Vero E6 cells were inoculated with cell-free suspensions of SARS-CoV-2 Beta (2x10^5^ PFU/mL in 500 µL) following illumination with 0 J/cm^2^, 15 J/cm^2^, and 90 J/cm^2^ of 425 nm light. At 3 hpi and 24 hpi, total RNA was extracted from inoculated cells for qRT-PCR analysis of N1 (A), N2 (B), and RNaseP (C) with the CDC RUO assay kit and TaqMan Fast Virus 1-Step Master Mix. Data presented are mean Ct +/- SEM (n = 4) for the N1, N2, and RNaseP probes at 3 hpi and 24 hpi. Statistical significance was determined via the Mann-Whitney ranked sum test and is indicated by * (p<0.05) and ** (p<0.01).

**Figure S3. 425 nm light does not expose viral RNA to RNaseI degradation.** Heat-inactivated (A) and active, replication-competent WA1 (B) were illuminated with 425 nm light and incubated with or without RNase and Triton X-100. RNA copies were determined via qRT-PCR. Data presented are the mean log_10_ RNA copy number +/- SEM (n = 4). Statistical significance was determined via the Mann-Whitney ranked sum test and is indicated by * (p<0.05). Statistical significance was determined relative to the 0 J/cm^2^ (-/-) group.

**Figure S4. 425 nm light illumination impairs SARS-CoV-2 spike-expressing pseudovirion entry to susceptible host cells.** Pseudovirions expressing WA1 or Omicron spikes were illuminated and evaluated for their entry to susceptible cells. Pseudovirions expressing WA1 (A) or Omicron (B) spikes were illuminated and evaluated for their entry to susceptible cells. Data presented are relative light units +/- SEM (n = 4) for WA1 or Omicron spike-expressing pseudovirions.
